# Convergent Evolution Associated with Habitat Decouples Phenotype from Phylogeny in a Clade of Lizards

**DOI:** 10.1371/journal.pone.0051636

**Published:** 2012-12-12

**Authors:** Shelley Edwards, Bieke Vanhooydonck, Anthony Herrel, G. John Measey, Krystal A. Tolley

**Affiliations:** 1 Applied Biodiversity Research Division, South African National Biodiversity Institute, Cape Town, South Africa; 2 Department of Botany and Zoology, University of Stellenbosch, Matieland, South Africa; 3 Department of Biology, University of Antwerp, Antwerp, Belgium; 4 UMR 7179 CNRS/MNHN, Département d’Ecologie et de Gestion de la Biodiversité, Paris, France; 5 Department of Zoology, Nelson Mandela Metropolitan University, Port Elizabeth, South Africa; British Columbia Centre for Excellence in HIV/AIDS, Canada

## Abstract

Convergent evolution can explain similarity in morphology between species, due to selection on a fitness-enhancing phenotype in response to local environmental conditions. As selective pressures on body morphology may be strong, these have confounded our understanding of the evolutionary relationships between species. Within the speciose African radiation of lacertid lizards (Eremiadini), some species occupy a narrow habitat range (e.g. open habitat, cluttered habitat, strictly rupicolous, or strictly psammophilic), which may exert strong selective pressures on lizard body morphology. Here we show that the overall body plan is unrelated to shared ancestry in the African radiation of Eremiadini, but is instead coupled to habitat use. Comprehensive Bayesian and likelihood phylogenies using multiple representatives from all genera (2 nuclear, 2 mitochondrial markers) show that morphologically convergent species thought to represent sister taxa within the same genus are distantly related evolutionary lineages (*Ichnotropis squamulosa* and *Ichnotropis spp*.; *Australolacerta rupicola* and *A. australis*). Hierarchical clustering and multivariate analysis of morphological characters suggest that body, and head, width and height (stockiness), all of which are ecologically relevant with respect to movement through habitat, are similar between the genetically distant species. Our data show that convergence in morphology, due to adaptation to similar environments, has confounded the assignment of species leading to misidentification of the taxonomic position of *I. squamulosa* and the *Australolacerta* species.

## Introduction

Convergent evolution is attributed to strong selection on a fitness-enhancing phenotype in response to local environmental conditions [1]. In reptiles, convergent evolution is found among species of *Anolis* lizards [2]–[4], amongst others, showing that similarities in environmental conditions and habitat use may elicit similar adaptive evolutionary responses by directional selection regardless of ancestry. Similar morphologies are also observed among distantly related rock-dwelling [5]–[8], burrowing [9]–[12], as well as arboreal lizards [13]–[15]. In each of these cases, adaptation is ascribed to selection on an animal’s body plan in order to optimize performance in a given habitat. For example, rock-dwelling species typically have flat heads and bodies that allow them to fit into narrow cracks, yet long forelimbs adapted for climbing [8], [16]. In contrast, some arboreal species that specialize on narrow substrates have short limbs and narrow, tall bodies [17]–[20].

Southern Africa has a diverse assemblage of macro-habitats, from tropical forest to desert, and ranges from sea level to more than 3000 m. This complexity at the macro scale is interwoven with a diversity of micro-habitat structure that includes different substrates and vegetation organization, and the heterogeneity at both scales may be a strong factor in producing high diversity and endemism of reptiles in the region [21], [22]. Indeed, many species are restricted and habitat specific at the micro scale (e.g. chameleons, cordylids), whilst others are apparent generalists (e.g. skinks). Morphological adaptation to this diversity in habitat structure should be reflected in phylogenies as lineages showing morphological convergence in species living in similar habitat structure, or divergence in species occupying different habitat structure.

Although phenotypic convergence is a common explanation for morphological similarity, such occurrences can be the result of phylogenetic history, chance and/or pre-existing constraints (‘exaptation’) rather than adaptation to similar environments [1]. Natural selection favors traits that increase fitness, even if the trait did not necessarily evolve in response to those selective pressures. While experimental conditions simulating environments can convincingly demonstrate whether natural selection drives convergence in morphological traits [23], it is more difficult to test convergence through adaptation to shared environments within a natural setting [24].Yet, repeated evolution of convergent phenotypes in divergent lineages inhabiting similar environments is often considered strong evidence of natural selection operating on morphological traits.

Here, we examine convergence of ecologically relevant phenotypic traits to habitat structure (cluttered and open vegetation) in a diverse group of lizards (Eremiadini, Lacertidae) from southern Africa. We predicted that ecologically relevant traits would converge in association with habitat similarity, regardless of evolutionary history. We postulated that species utilizing cluttered habitats would have relatively slender bodies and short limbs compared to species utilizing open habitats, to allow for efficient movement through the cluttered matrix [17], [25], [26]. To test this hypothesis, we investigated the evolutionary relationships in the Eremiadini using a multi-locus phylogenetic approach, in combination with principal components analysis and hierarchical clustering for morphological data on traits that are considered ecologically relevant to lizards [8], [27]. The clusters were then compared *a priori* to habitat structure to examine occurrences of convergence in morphology between species.

## Methods

### Sampling

Taxa chosen for the study were genera from the southern African clade of the lacertid lizards from the tribe Eremiadini (five genera out of 20 total genera in Eremiadini). Samples for the genetic analyses were obtained either from field trips conducted by myself or from samples, collected by various researchers, housed in the collection at the South African National Biodiversity Institute. Some of the individuals sampled have been sequenced previously for the 16S and RAG1 genes, and accession numbers and references are provided in Table S1. Samples for the morphometric analyses included measurement of live lizards during field work, as well as voucher specimens housed at the Port Elizabeth Museum (PEM), the Ditsong Museum (TM) and the Ellerman Collection at Stellenbosch University.

### Ethics Statement

Ethics clearance was obtained from University of Stellenbosch (permit no. 11NP-EDW01) and South African National Biodiversity Institute (permit no. 002/10), permitting the collection and handling of the lizards, as well as the sampling of tail tissue.

### Laboratory Protocols

Genomic DNA was isolated from the tail or liver tissue preserved in 95–100% ethanol according to standard procedures involving a proteinase K digestion followed by salt-extraction [28]. Standard PCR procedures were utilized to amplify two mitochondrial (16S and ND4) and two nuclear genes (RAG1 and KIAA-2018). The nuclear genes were chosen because these genes have been shown to evolve at a rate that may allow high confidence in both the terminal and the deeper nodes [29], [30]. For the mitochondrial genes, the primer pairs ND4 and tRNALeu [31], and L2510 and H3080 16S rRNA [32] were used to amplify the ND4 and 16S genes, respectively. The primers RAG1-F0 and RAG1-R1 [33], and KIAA2018-F1 and KIAA2018-R2 [30] were used to amplify the nuclear RAG1 and KIAA-2018 genes, respectively.

Amplification of the four genes was carried out with ∼25–50 ng/µl genomic DNA and a 25 µl reaction containing a thermophilic buffer (50 mM KCl, 10 mM Tris–HCl, pH 9.0), 1.5 mM MgCl2, 0.2 µM of each primer, 0.2 mM dNTPs, and 0.025 U/l Taq polymerase. Cycling profile for 16S, ND4 and KIAA-2018 genes included an initial denaturing step at 94°C for 4 minutes, followed by 35 cycles of 94°C for 30 s, 50–55°C for 30 s, and 72°C for 45 s, with a final extension at 72°C for 8 min. The amplification of the RAG1 gene region involved a step-down procedure [34]. The PCR products were sent to Macrogen Corp. (Seoul, Korea) for sequencing using the forward primers in all cases. Sequences were aligned in BioEdit Sequence Alignment Editor v. 7.0.5.2 [35]. All sequences have been deposited in EMBL-Bank (see Table S2 for all voucher information, with corresponding EMBL-Bank accession numbers).

### Genetic Analyses

We first analyzed the mitochondrial (16S vs. ND4) and nuclear (RAG1 vs. KIAA2018) datasets separately to ensure that there was no conflict in the markers within each genome, using a partition homogeneity test [36], [37] in PAUP* v4.0b10 [38]. The two mitochondrial and the two nuclear genes were not incongruent, so the partition homogeneity test was run again (nuclear vs. mitochondrial) to ensure that there was no conflict between the two genomes. Phylogenetic trees were constructed of the 1) mitochondrial gene dataset (Fig. S1), 2) the nuclear gene dataset (Fig. S1) and 3) the combined total evidence dataset (Fig. S2). The saturation of the codon positions was assessed using the program Dambe v.5.2.65 [39]. Even though the third codon position of the ND4 gene was found to be saturated, it was not excluded from the analyses, but rather it was coded as a separate partition. Two individuals of *Heliobolus lugubris* were used as the outgroup, as it is within the sister clade to the southern African radiation within Eremiadini [33], [40]. Sequence divergences were determined by estimating the uncorrected p-distances between and within species using the program MEGA v.4 [41].

**Figure 1:**
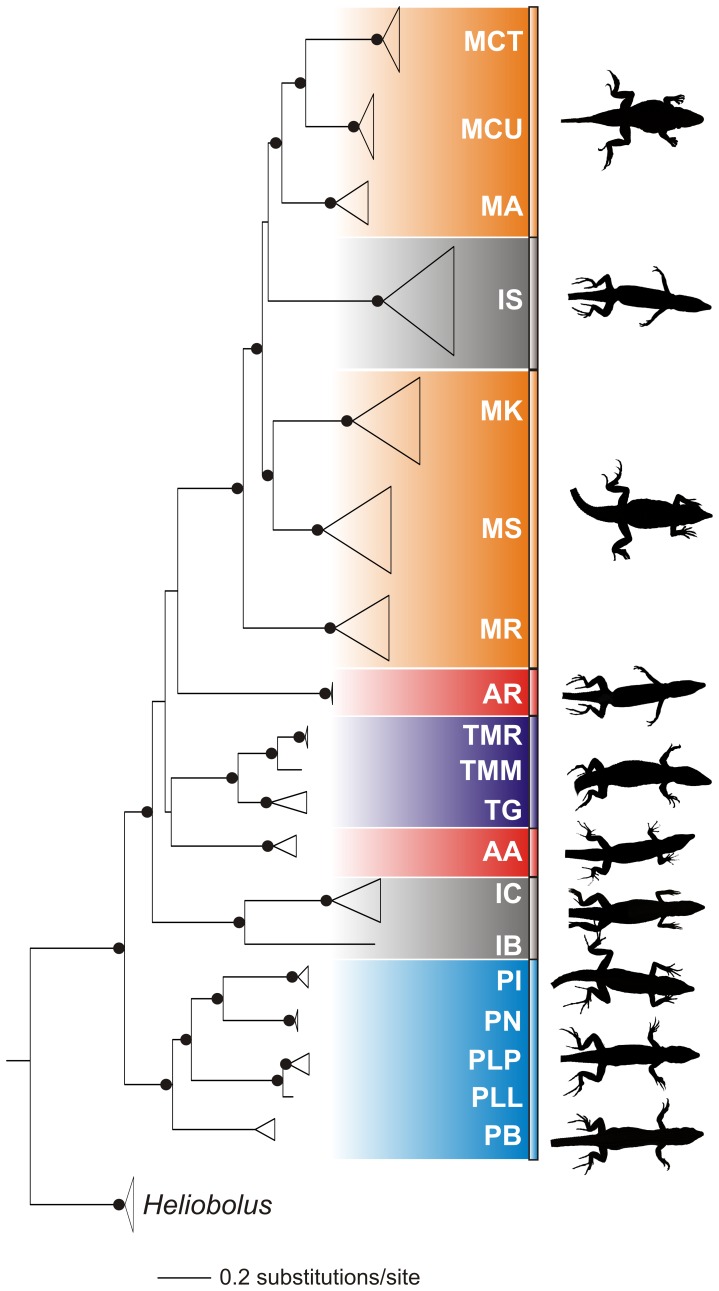
Phylogeny of the southern African lacertids. Phylogenetic reconstruction (left) using Bayesian inference (BI) of the southern African radiation of the lacertid subfamily Eremiadini based on the combined partial *16S*, *ND4*, *RAG1* and *KIAA* gene regions and inferred by BI and maximum likelihood (ML). Nodes with filled circles indicate BI posterior probabilities ≥0.95 and ML bootstrap values ≥75%. Representatives of the body shapes for each general clade are included (right) to show differences in bauplan of the main genetic clades. Key to the color coding for genera and species abbreviations: *Australolacerta* (red): AA = *Australolacerta australis*, AR = *A. rupicola*; *Ichnotropis* (gray): IB = *Ichnotropis bivittata*, IC = *I. capensis*, IS = *I. squamulosa*; *Meroles* (orange): MA = *Meroles anchietae*, MCT = *M. ctenodactylus*, MCU = *M. cuneirostris*, MK = *M. knoxii*, MS = *M. suborbitalis*; *Pedioplanis* (light blue): PB = *Pedioplanis burchelli*, PI = *P. inornata*, PLL = *P. lineoocellata lineoocellata*, PLP = *P. l. pulchella*, PN = *P. namaquensis*; *Tropidosaura*(blue): TG = *Tropidosaura gularis*, TMM = *T. montana montana*, TMR = *T. m. rangeri*.

Two different algorithms were utilized to obtain phylogenetic trees (Figs. 1 and S1). Bayesian inference (BI) was performed using the program MrBayes v.3.1.0 [42], [43]. Priors in MrBayes were set according to the evolutionary model which best fits the dataset using the program MrModeltest v.3.6 [44], and uniform priors were kept for all other parameters. The MCMC were run with 2 parallel runs for 10 million generations each, with trees sampled every 1000 generations. The number of generations to discard as burn-in was determined by examining the number of generations 1) at which the standard deviation of split frequencies stabilized (at less than 0.001), 2) at which the log-likelihood tree scores reached stationarity, and 3) the effective sample sizes (ESS) of all parameters which were ≥600 (using the program Tracer v.1.5 [45]). A 50% majority rule tree was constructed with the burn-in excluded using the “sumt” command in MrBayes, and nodes with ≥0.95 posterior probability were considered supported. A Shimodaira–Hasegawa (SH) test [46], [47] was performed to compare the consensus tree with a tree where *I. squamulosa* was constrained to be closely related to *Ichnotropis*.

A partitioned maximum likelihood (ML) analysis was also run in RAxML v.7.2.8 [48], at the CIPRES Science Gateway (www.phylo.org/sub_sections/portal/) using the same partitions as the Bayesian analysis, a GTR+I+G model of evolution, and automatic halting of bootstrapping [48], [49].

### Characterization of Habitat

Two broad habitat types (open and cluttered) were defined for our analysis based on the general characteristics of vegetation structure associated with each species sampled. Open habitat lacks vegetation completely (i.e. dunes) or is sparsely vegetated, and mainly characterized by open sand, gravel or rock patches briefly interspersed with bushes or grass tufts. A cluttered habitat is densely vegetated (i.e. with low vegetation such as grasses, sedges and restios, with an abundance of bushes in various sizes), with intermittent open patches (Fig. S3).

### Morphometric Analyses

Body length (snout-vent length; SVL) and biometric characters on the head, hind limbs and fore limbs were measured externally using digital calipers for each individual. Measurements on the crania that related to the length of the head have seldom been investigated in lizards in terms of habitat openness, however the height and width of the crania have been linked to the use of specific refuges in cluttered environments (e.g. crevices in rocky habitats [8]). Measurements taken on the head were: head length (HL) from snout-tip to the back of the parietal bone, head width (HW) measured as the widest part of the head, head height (HH) measured as the height from the top of the interparietal scale to the bottom of the lower jaw (including muscles), lower-jaw length (LJL), coronoid to snout-tip length (CT), and quadrate to snout-tip length (QT). Limb measurements were taken of both the hind- and fore-limbs, as cluttered habitats have been cited as a factor in limb reduction [26], [50], and longer limbs may be necessitated by an open habitat for higher sprint speeds, in order to effectively escape predators [17], [25], [26]. Measurements taken on the limbs were as follows: the femur length (FM), tibia length (TB), humerus length (HM) and radius length (RD). Other body dimensions measured were body height (BH) and body width (BW). Accession numbers for each individual and number of individuals measured for each species is detailed in Table S2.

Hierarchical clustering of the species was performed in the program R Studio v.0.94.84 [51], to identify morphological clusters. The mean value per species (17 species) of each size-regressed measurement (12 measurements) was calculated (package: ‘base’, function: ‘mean’ [50]) and the mean values per species for each measurement were regressed onto the mean snout-vent length (SVL) using a linear model to eliminate the effect of size (package: ‘stats’, functions: ‘lm’ and ‘resid’ [52]). Hierarchical clustering of the residual distances was performed (package: ‘pvclust’, function: ‘pvclust’ [53]) in which the distance matrix was calculated using the “correlation” option, the clustering dendrogram was constructed using the “complete” option, and support values for the nodes were estimated using 1000 bootstrap replicates.

**Figure 2:**
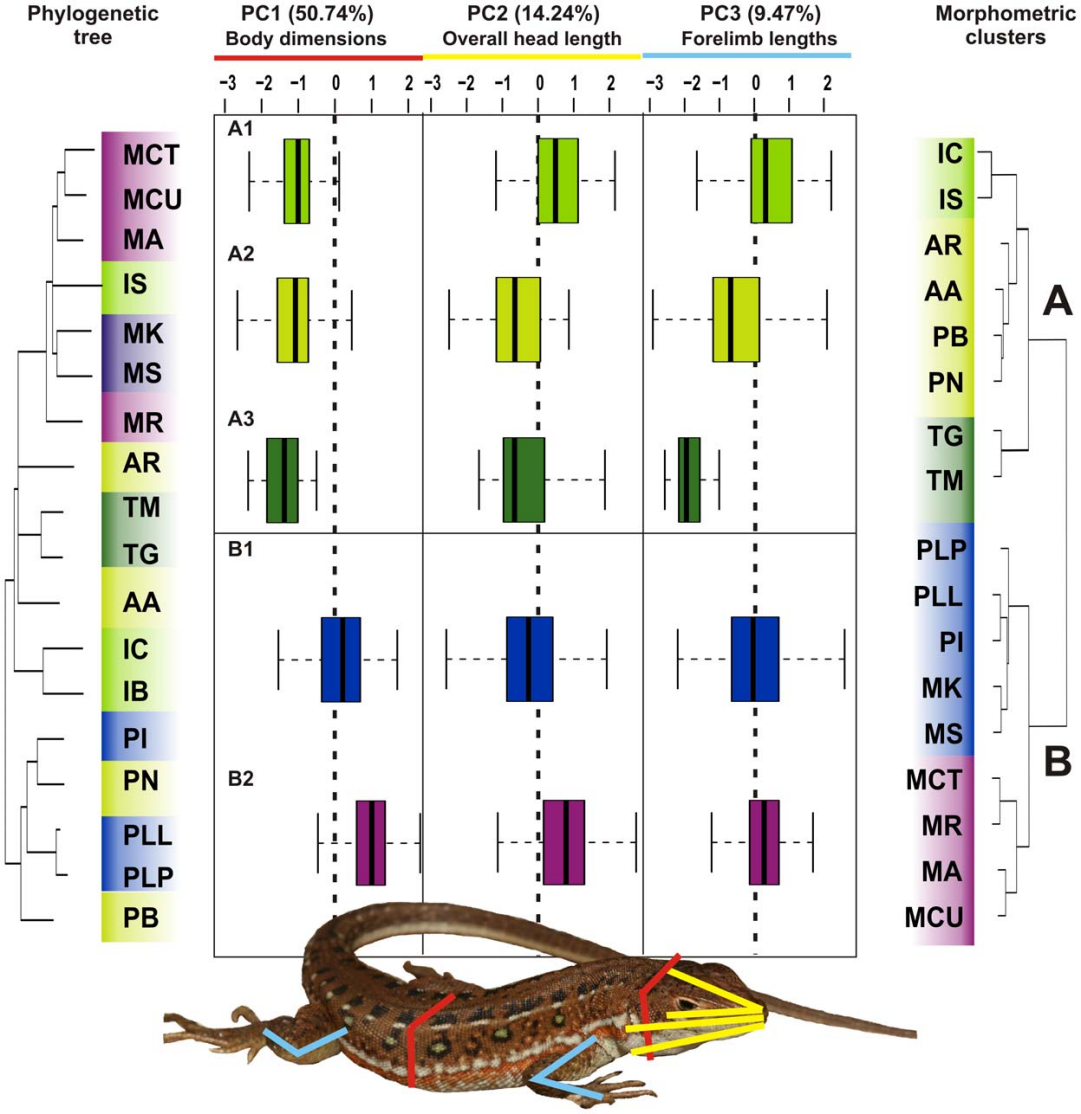
Clustering and principal components analysis of morphological markers. Boxplots of the first three principal component axes (center) for each morphological group (A, B) retrieved by hierarchical clustering (shown right). Positive values of the PC axes indicate larger body dimensions, whilst negative values indicate smaller body dimensions. Morphological groupings are shaded as follows: A1 =  bright green, A2 =  lime green, A3 =  green, B1 =  blue, B2 =  purple. The phylogenetic tree (left) is color coded by species according to its morphological group membership. Morphological measurements are shown on lizard schematic, and line colors correspond to sets of original variables that loaded onto each PC (PC1 =  red, PC2 =  yellow, PC3 =  light blue). Percentage of variation contributed to each PC axis is given. Key to the species abbreviations as in Fig. 1.

To examine trait differences among the morphological groupings obtained in the hierarchical clustering, a principal components analyses (PCA) on the residuals was performed in the program SPSS v.15 (SPSS, Inc.). Varimax rotation was used and three principal components (PC) with eigenvectors greater than 1 were extracted, which accounted for *ca*. 74.45% of the total variance (Table 1). The KMO test indicated sampling was adequate (i.e. in excess of 0.5), all communalities were high (*i.e*. in excess of 0.5) suggesting that all variables were reliable contributors to the analysis, there were sizeable correlations between all original variables, and low correlations in the residual correlation matrix [54]. The three PC’s extracted (Table 1) loaded highest with body and head width (PC1), head lengths (PC2), and limbs (PC3). Boxplots (Figs. 2 & S4) were constructed using the PC scores for these same groups (package: ‘stats’, function: ‘boxplot’ [51]). Analysis of variance (ANOVA) was carried out on the three principal components extracted with the morphological cluster as the fixed factor (package: ‘stats’, function: ‘anova’ [51]).

**Table 1:** Principal components analysis loadings of size-regressed measurements.

Residuals	PC1	PC2	PC3
**Body width (BW)**	**0.89**	0.07	0.03
**Head width (HW)**	**0.79**	0.32	0.27
**Body height (BH)**	**0.77**	0.14	−0.02
**Head height (HH)**	0.60	0.53	0.21
**Lower jaw length (LJL)**	0.27	**0.81**	0.10
**Quadrate-Tip length (QT)**	0.29	**0.78**	0.29
**Head length (HL)**	0.40	**0.76**	0.23
**Coronoid-Tip length (CT)**	−0.07	**0.70**	0.27
**Radius length (RD)**	0.02	0.23	**0.88**
**Humerus length (HM)**	0.02	0.21	**0.87**
**Tibia length (TB)**	0.52	0.27	**0.66**
**Femur length (FM)**	0.58	0.30	0.59
**% variance**	50.74	14.27	9.47
**F-value**	430.19 (***)	2.60 (ns)	15.77 (***)

Principal components analysis of size-regressed measurements, with loadings of each measurement for the three axes that had eigenvalues >1.0.Characters that loaded most strongly with each principal component are in bold. F-values from the analysis of variance between two main morphological clusters are shown. ***P<0.001; ns-not significant.

## Results

The combined mitochondrial and the nuclear topologies (BI and ML) were congruent (Figs. 1 and S1) and largely consistent with previous work [33], [40]. Our data however shows two notable exceptions due to the inclusion of additional taxa (*Ichnotropis spp.* and *Australolacerta spp.*), both of which suggest that factors independent of ancestry are driving morphological evolution in the Eremiadini. Firstly, the two species of *Australolacerta* are separate evolutionary lineages, and form part of a deep basal polytomy at the generic level (Figs. 1 & S1), despite the ecological and morphological similarities that were used to place them in the same genus (Fig. 2 [55]). Secondly, the phylogeny shows that *Ichnotropis squamulosa* shares its most recent ancestry with members of the genus *Meroles* (Figs. 1 & S1), rather than with species in the morphologically similar genus *Ichnotropis* (Fig. 2), leading to a misclassification at the generic level. There was a significant difference between the Bayesian consensus tree and the tree where *I. squamulosa* was constrained as part of *Ichnotropis* (SH test: P<0.01). Sequence divergences also show that *Ichnotropis squamulosa* is highly divergent from other *Ichnotropis* species examined (16S: 10.96±2.27%, ND4∶21.80±2.62%, RAG1∶5.09±0.88%, KIAA: 3.38±0.1%). In both cases, convergence in bauplan is coupled to traits associated with body/head width and limb dimensions (Fig. 2).

The phylogenetic analyses show that the two species, *A. australis* and *A. rupicola*, are separate evolutionary lineages, and form a basal polytomy with all other Eremiadini genera except *Meroles*. The SH test was not performed with *Australolacerta* constrained as monophyletic group, due to the unresolved relationship between the two species. The sequence divergence between these lineages were high (16S: 9.55±2.08%; ND4∶22.69±1.60%; RAG1∶3.74±0.76%; KIAA: 1.90±0.47%), consistent with generic divisions in southern African Lacertidae (16S: 7.57±1.38%; ND4∶21.21±1.33%; RAG1∶4.07±0.54%; KIAA: 2.84±0.60%, this study) as well as others (combined RAG1 & C-MOS: 1.40% between *Archaeolacerta* and *Zootoca*, [33]). Due to the high sequence divergences, we suggest that they have been incorrectly placed together in a single genus due to their similar body plans.

The adaptive nature of convergence in Eremiadini is demonstrated by the significant association of ecologically relevant traits and habitat structure. Hierarchical clustering of morphological features resulted in two major clusters that correspond to A) cluttered and B) open habitats (Fig. 2). These morphological clusters do not correspond to the evolutionary history of these taxa, but instead are significantly different with respect to sets of ecologically relevant characteristics related to habitat structure. Each cluster was further subdivided into either three (Cluster A: A1, A2 and A3) or two (Cluster B: B1 and B2) subclusters. Some of the subclusters can be linked to particular microhabitats within a cluttered or open habitat. For example, Cluster B2 species are dune-dwelling, whilst species of Cluster A2 and A3 are rupicolous.

Multivariate analyses (principal components analysis and analysis of variance) indicate that the two morphological clusters differ significantly in terms of body/head slenderness (PC1: F = 430.19, p<0.001, 50.74% of the variation; Table 1), with species inhabiting cluttered habitats being slender and more elongate compared to those in more open habitats (Figs. 1 & S5). The two morphological clusters did not differ significantly for the second principal component (PC2: F = 2.60, p = 0.11, 14.24% of the variation) that loaded positively with most head measurements, particularly lengths (Table 1). An exception is that dune-dwelling species (B2) have significantly longer heads compared to clusters B1 (F = 98.86, p<0.0001) and A2 (F = 24.73, p<0.0001) (Table 2). The two clusters differed significantly for PC3 (F = 15.77, p<0.001; 9.47% of the variation), however this may be due to the relatively shorter forelimbs of *Tropidosaura* (A3).

**Table 2:** Analysis of variance (ANOVA) results for morphological clusters.

PC1		Df	Sum-Sq	Mean-Sq	F-value	P	PC2		Df	Sum-Sq	Mean-Sq	F-value	P	PC3		Df	Sum-Sq	Mean-Sq	F-value	P
A1	A2	1	0.67	0.67	1.54	0.22	A1	A2	1	37.50	37.50	56.04	***<0.001***	A1	A2	1	28.32	28.32	34.28	***<0.001***
	A3	1	1.84	1.84	6.64	***0.01***		A3	1	10.92	10.92	17.71	***<0.001***		A3	1	61.63	61.63	108.44	***<0.001***
	B1	1	63.95	63.95	147.48	***<0.001***		B1	1	27.60	27.60	36.36	***<0.001***		B1	1	6.91	6.91	7.09	***0.01***
	B2	1	139.27	139.26	409.44	***<0.001***		B2	1	1.90	1.90	2.78	0.10		B2	1	1.16	1.16	2.41	0.12
A2	A3	1	0.71	0.71	1.30	0.26	A2	A3	1	0.39	0.39	0.49	0.48	A2	A3	1	20.25	20.25	24.28	***<0.001***
	B1	1	89.80	89.80	181.98	***<0.001***		B1	1	6.21	6.21	7.79	***0.01***		B1	1	17.64	17.64	17.15	***<0.001***
	B2	1	175.14	175.14	384.49	***<0.001***		B2	1	73.35	73.35	97.15	***<0.001***		B2	1	25.77	25.77	42.71	***<0.001***
A3	B1	1	36.02	36.02	77.89	***<0.001***	A3	B1	1	0.44	0.44	0.54	0.46	A3	B1	1	50.91	50.91	50.98	***<0.001***
	B2	1	73.60	73.60	197.33	***<0.001***		B2	1	18.76	18.76	24.73	***<0.001***		B2	1	58.64	58.64	156.77	***<0.001***
B1	B2	1	44.42	44.42	100.31	***<0.001***	B1	B2	1	77.69	77.69	98.86	***<0.001***	B1	B2	1	3.45	3.45	4.07	***<0.001***

Analysis of variance (ANOVA) results for morphological clusters (A1, A2, A3, B1 and B2; as in Fig. 2). Significant differences (P<0.05) are indicated in bold, italic font. PC = principal component, Df = degrees of freedom, Sum-Sq = sum of squares value, Mean-Sq = mean sum of squares value, P = significance value.

## Discussion

Whilst morphological characters are traditionally used to define species, descriptions that incorporate multidisciplinary approaches, including morphological, genetic, behavioral and ecological aspects, are typically better informed (*e.g.* [56]). Our data shows that among the morphological similarities upon which taxonomic classifications for Eremiadini are based [57], some are the result of convergence due to habitat structure and not shared ancestry. We show that convergent evolution of morphological characters has led to genetically distant, but partially sympatric (*Ichnotropis* spp.) and parapatric (*Australolacerta* spp.) species being considered as sister taxa. Such examples of misclassification due to phenotypic similarities between species are increasingly familiar, suggesting that morphological adaptation in response to similar environments is pervasive, rather than exceptional. Even what might appear to be obvious cases of shared evolutionary history based on morphology, have turned up surprising developments revealing incorrect classifications at the generic level (e.g. geckos of the genera *Pachydactylus*/*Elasmodactylus* [58]; chameleons of the genera *Archaius*/*Rieppeleon* [59]).

Convergence in phenotype can be the result of random evolutionary change [1], however the observed morphological convergence in the southern African lacertids suggests adaptation to particular environments. The high genetic divergence between morphologically and ecologically similar species suggests that vegetation density (i.e. habitat clutter) is a major driving force in the evolution of phenotypic diversity in these lizards, irrespective of ancestry. Within the lacertid lizards, the phylogenetic position of species inhabiting particular environments (i.e. xeric or mesic environments) was investigated previously [33], and a unique monophyletic trend from mesic to xeric species within the Lacertidae could not be demonstrated, despite previous morphological phylogenies which showed this trend [57], [60]. With the comparison of the molecular tree to the broad environmental categories, it was suggested that there are multiple origins of xeric-adapted species within Eremiadini. However, here we show that morphology of a lizard is likely to be driven by its microhabitat, with less association to broad scale biome features. In fact, for many reptiles, geographic proximity influences phylogenetic position (e.g. [13], [61]) making it unsurprising that a link exists between broad scale environmental classifications and phylogenetic position. For example, within *Meroles*, *M. anchietae* and *M. cuneirostris* are in the same clade, have a similar body plan and both inhabit a xeric environment. However, the lack of phylogenetic independence means that similarities due to a common ancestor which inhabited the xeric region prior to diversification cannot be ruled out. Conversely, *M. reticulatus* is not within the same clade as *M. anchietae* and *M. cuneirostris*, but the bauplans of all three species are similar suggesting a separate origin of this morphology due to similarity in microhabitat (open habitat) within the xeric macrohabitat.

Whilst the morphological clusters were significantly different with respect to overall body slenderness (PC1) and linked to habitat openness, the lack of a significant difference for PC2 (Table 1) indicates that head shape is driven by factors other than habitat structure such as diet or sexual selection (e.g. [62], [63]). Convergence in head shape within the dune-dwelling species (B2) may be as a result of their preference to sand-dive or to utilize burrows, both of which are behavioral adaptations for predator avoidance and thermoregulation [27], [64]. The *Ichnotropis* (A1) head dimensions are not significantly different from the dunes cluster (B2) (F = 2.78, p = 0.10), and this could be due to a propensity for digging burrows for shelter and reproduction [65], thereby evolving the same relative head morphology [65]. Another possibility is that *Ichnotropis* may have a similar diet to the sand-dwelling species, which may be driving the similarity in head shape [65].

In terms of limb lengths, the two morphological clusters were significantly different (PC3), in particular because of the short limbs in *Tropidosaura.* The shorter forelimbs in conjunction with their slender bodies may allow *Tropidosaura* to optimize maneuvering performance while negotiating cluttered vegetation (e.g. [17], [27]), whereas the long limbs of the *Ichnotropis* spp. (A1) and those inhabiting more open habitats (B1 and B2) should increase sprint performance (e.g. [26], [27], [66]–[68]). Relative forelimb and hindlimb dimensions, however, need to be investigated in conjunction with substrate type and structure, as opposed to habitat structure, in order to better understand the evolution of limb dimensions in Eremiadini.

Although sub-sets of taxa from *Meroles* and *Ichnotropis* were investigated as part of higher level lacertid phylogenies, the placement of *I. squamulosa* within *Meroles* was not identified previously due to the inclusion of only a single *Ichnotropis* (*I. squamulosa*) and various *Meroles* (*M. knoxii, M. suborbitalis* or *M. ctenodactylus*) in those analyses [33], [40], [69]. Despite their placement in the phylogeny, *I. capensis* and *I. squamulosa* do not differ significantly morphologically, and cluster together when body dimensions, head measurements and limbs measurements are investigated. Both of these species possess more slender bodies relative to *Meroles.* In addition, they share characters not possessed by *Meroles* (rough scales and the absence of a nuchal collar). Because these two species have partially sympatric distributions, their overlapping niche might explain the observed morphological similarities. For example, limb dimensions could reflect adaptation to substrate type, while head shape similarities could reflect adaptation to similar diets. Although neither have a nuchal collar, this is also absent in other *Meroles* (*i.e. M. anchietae*), as well as other lacertids (*e.g*. *Tropidosaura*). Thus, the presence/absence of the collar is unlikely to be a synapomorphy (Fig. S1). Similarly, the presence of a gular fold (similar to a nuchal collar, but does not extend all the way around the head) does not appear to be a character than can be used to indicate shared ancestry (Fig. S1). The other characteristic feature that has linked these species in the past is the presence of rough (strongly keeled) scales. However, this is also not a synapomorphy as other lizards and even lacertids (*e.g. Tropidosaura*) are known to have rough scales suggesting shared scale micro-ornamentation is not an indication of a shared ancestry in lacertid lizards but rather related to microhabitat use [70].

There are several interesting implications of the placement of *I. squamulosa* within *Meroles*, rather than *Ichnotropis*. Sympatry often leads to competition for resources particularly between closely related species. *Ichnotropis squamulosa* is sympatric with *I. capensis* in the northern regions of its distribution, but is allopatric with all *Meroles*. Whilst *Meroles* are primarily sand-dwellers, *Ichnotropis* are classified as terrestrial [71], with a propensity for sandy habitats in mesic and arid savannah [65]. The reproductive cycles of *I. squamulosa* and *I. capensis* are not concordant [65], [72], [73], which is thought to prevent interspecific competition [72], [73]. Both species are considered to be annual breeders, although the breeding times are staggered [73], and life-spans are unusually short for lacertid lizards. *Ichnotropis squamulosa* lives approximately eight to nine months, mating in late summer and hatchlings appear in spring [65], [73]. *Ichnotropis capensis* may live only marginally longer (13–14 months), mating in spring with hatchlings appearing in late summer [65], [73]. It has been suggested that this staggered reproductive pattern arose to prevent interspecific competition between closely related species [74]. However, because these species are not closely related, this shared life-history trait cannot be associated with a reduction of competition between sister taxa, but rather suggests an independent evolution of a similar but temporally disjunct reproductive strategy. The reasons for this are not clear, particularly because *I. squamulosa* still exhibits the same reproductive strategy in regions where the two species are not sympatric (e.g. in Upington, South Africa [73]) suggesting that the staggered reproduction of the two species is not driven by interspecific competition.

Morphological adaptation to a particular microhabitat may confer a greater fitness to individuals through their performance (for a review see [75]).We show that habitat openness determines the morphological shape of southern African lacertid species and we expect that these differences in morphology will, in turn, be associated to performance differences between the species. Those species adapted to open dunes may be better sprinters than those inhabiting cluttered rocky environments, whilst the rock-dwellers may be better climbers than sand dwellers. A closer investigation into associations between body and limb shape and performance in southern African lizards is needed to understand the functional implications of the morphological shape differences in southern African lacertid lizards.

## Supporting Information

Figure S1 Phylogenetic trees of the southern African radiation of the lacertid subfamily Eremiainae based on the partial (A) *16S*, (B) *ND4*, (C) *RAG1* and (D) *KIAA* gene regions and inferred by BI. Sample numbers are indicated at terminal tips, and species names are given (right). Posterior probabilities ≥0.95 are above the nodes. Key to the species abbreviations: *Australolacerta* (red): AA = *Australolacerta australis*, AR = *A. rupicola*; *Ichnotropis* (gray): IB = *Ichnotropis bivittata*, IC = *I. capensis*, IS = *I. squamulosa*; *Meroles* (orange): MA = *Meroles anchietae*, MCT = *M. ctenodactylus*, MCU = *M. cuneirostris*, MK = *M. knoxii*, MS = *M. suborbitalis*; *Pedioplanis* (light blue): PB = *Pedioplanis burchelli*, PI = *P. inornata*, PLL = *P. lineoocellata lineoocellata*, PLP = *P. l. pulchella*, PN = *P. namaquensis*; *Tropidosaura* (blue): TG = *Tropidosaura gularis*, TMM = *T. montana montana*, TMR = *T. m. rangeri*.
(TIF)

Figure S2 Phylogenetic tree of the southern African radiation of the lacertid subfamily Eremiainae based on the combined partial *16S*, *ND4*, *RAG1* and *KIAA* gene regions and inferred by BI and ML (Bayesian topology shown). Sample numbers are indicated at terminal tips, and species names are given. Posterior probabilities ≥0.95 are above the nodes and bootstrap values ≥75% are below nodes. Filled stars next to species names indicate presence of both a gular fold and a nuchal collar in the species, open stars indicate presence of nuchal collar only, filled circles indicate presence of gular fold only.
(TIF)

Figure S3 Photographs of cluttered (A) and open habitat (B), as examples of the two habitat categories defined for this study (Photos by SE).
(TIF)

Figure S4 Hierarchical clustering of size-regressed morphological measurements, with “approximately unbiased” support values above the nodes. Support values ≥0.95 are considered supported. For key to cluster abbreviations see Figure 2 and key to the species abbreviations see Figure S2.
(TIF)

Figure S5 Scatterplots of the principal components analysis (PCA) scores for the first and second (bottom), and first and third (top) principal component axes. Colors of the symbols correspond to the hierarchical clustering: green = A1, light green = A2, dark green = A3, light blue = B1, dark blue =  B2. Boxplots next to axes show the mean and 95% confidence intervals of each morphological clusterfor each PC axis, and label abbreviations as in Figure 2. Boxplots of PC1 below the scatterplots, PC2 are bottom-left and PC3 are top-left. Divisions for the boxplots are indicated by the color and at the axis.
(TIF)

Table S1 List of specimens used in the phylogenetic analyses with genus and species names, ID numbers, Museum accession ID numbers and EMBL accession numbers for each gene.
(DOCX)

Table S2 List of specimens used in morphometric analyses, genus and species names, ID numbers from either the Ditsong museum (TM), Port Elizabeth Museum (PEM), or field trips.
(DOCX)
