# Supplementary figures and images for: Convergent Evolution Associated with Habitat Decouples Phenotype from Phylogeny in a Clade of Lizards
Source: PLoS One. 2012 Dec 12;7(12):e51636. doi: 10.1371/journal.pone.0051636 (PMC3520956; doi:10.1371/journal.pone.0051636)

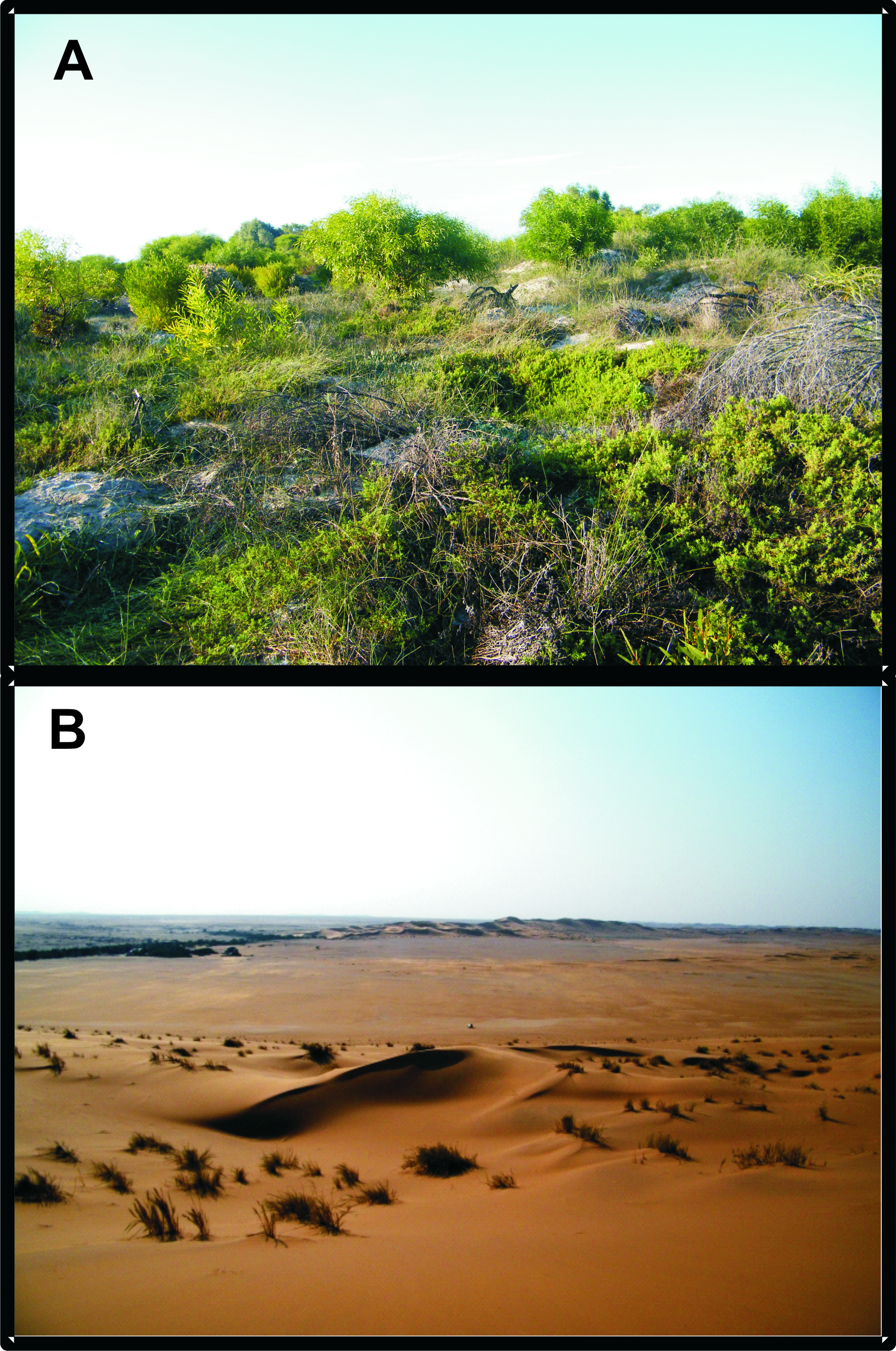

Supplement: Figure S3 — Photographs of cluttered (A) and open habitat (B), as examples of the two habitat categories defined for this study (Photos by SE). (TIF) [file pone.0051636.s003.tif]

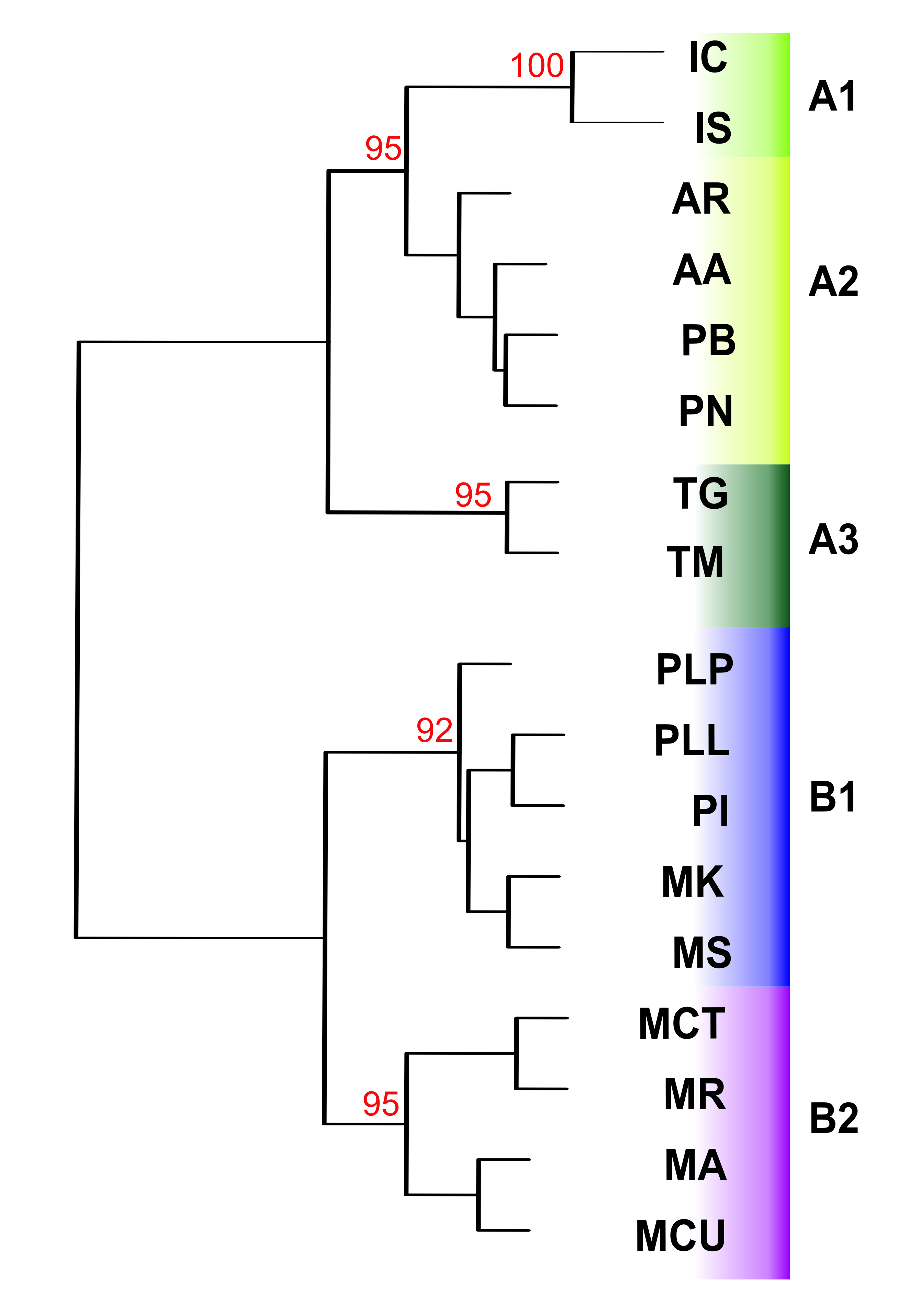

Supplement: Figure S4 — Hierarchical clustering of size-regressed morphological measurements, with “approximately unbiased” support values above the nodes. Support values ≥0.95 are considered supported. For key to cluster abbreviations see Figure 2 and key to the species abbreviations see Figure S2. (TIF) [file pone.0051636.s004.tif]

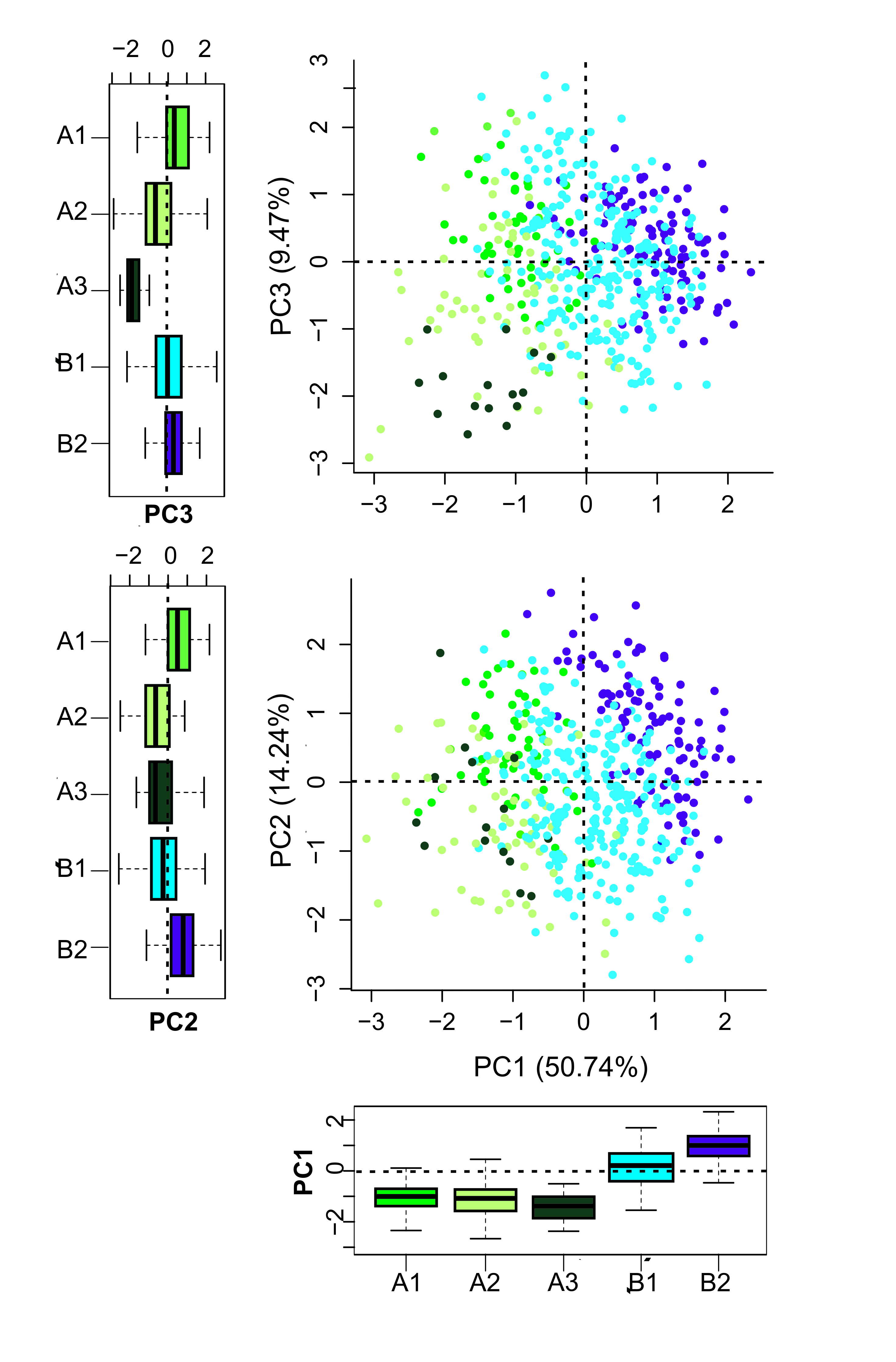

Supplement: Figure S5 — Scatterplots of the principal components analysis (PCA) scores for the first and second (bottom), and first and third (top) principal component axes. Colors of the symbols correspond to the hierarchical clustering: green = A1, light green = A2, dark green = A3, light blue = B1, dark blue = B2. Boxplots next to axes show the mean and 95% confidence intervals of each morphological clusterfor each PC axis, and label abbreviations as in Figure 2. Boxplots of PC1 below the scatterplots, PC2 are bottom-left and PC3 are top-left. Divisions for the boxplots are indicated by the color and at the axis. (TIF) [file pone.0051636.s005.tif]
