# Supplementary material for: Convergent Evolution Associated with Habitat Decouples Phenotype from Phylogeny in a Clade of Lizards
Source: PLoS One. 2012 Dec 12;7(12):e51636. doi: 10.1371/journal.pone.0051636 (PMC3520956; doi:10.1371/journal.pone.0051636)
Supplement: Table S1 — List of specimens used in the phylogenetic analyses with genus and species names, ID numbers, Museum accession ID numbers and EMBL accession numbers for each gene. (DOCX) [file pone.0051636.s006.docx]

**Table S1: List of specimens used in the phylogenetic analyses with genus and species names, ID numbers, Museum accession ID numbers and EMBL accession numbers for each gene.**

| **Genus** | **Species** | **SANBI Herpbank Accession ID** | **Museum Accession ID*** | **EMBL accession number for**  **16S** | **EMBL accession number for ND4** | **EMBL accession number for RAG1** | **EMBL accession number for KIAA** |
| --- | --- | --- | --- | --- | --- | --- | --- |
| *Australolacerta* | *australis* | GW08 | — | HF547772 | HF547725 | HF547691 | HF547651 |
|  |  | MH0531 | — | DQ871152^$^ | HF547726 | DQ871208^$^ | HF547652 |
| *Australolacerta* | *rupicola* | MCZ38869 | MCZ38869 | HF547773 | HF547727 | HF547692 | HF547653 |
|  |  | MCZ38874 | MCZ38874 | HF547774 | HF547728 | HF547693 | HF547654 |
| *Heliobolus* | *lugubris* | MCZ37870 | MCZ37870 | DQ871141^$^ | HF547729 | DQ871199^$^ | — |
|  |  | MCZ37894 | MCZ37894 | DQ871142^$^ | HF547730 | DQ871200^$^ | HF547655 |
| *Ichnotropis* | *bivittata* | KTH09-075 | MBUR2074 | HF547775 | HF547731 | HF547694 | HF547656 |
| *Ichnotropis* | *capensis* | AMB6001 | — | DQ871148^$^ | HF547732 | DQ871206^$^ | HF547657 |
|  |  | AMB6067 | CAS209602 | DQ871149^$^ | HF547733 | DQ871207^$^ | HF547658 |
|  |  | WP031 | — | — | HF547734 | HF547695 | HF547659 |
| *Ichnotropis* | *squamulosa* | FP264B | — | — | HF547735 | HF547696 | — |
|  |  | MB21340 | — | — | — | HF547698 | HF547661 |
|  |  | RSP373 | — | HF547777 | HF547737 | HF547699 | HF547662 |
|  |  | SVN362 | — | HF547776 | HF547736 | HF547697 | HF547660 |
|  |  | WP122 | — | — | — | HF547700 | HF547663 |
|  |  | WP125 | — | HF547778 | HF547738 | HF547701 | — |
| *Meroles* | *anchietae* | PEMR17286 | PEMR17286 | HF547779 | — | — | — |
|  |  | WP928 | — | HF547781 | HF547740 | HF547703 | — |
|  |  | WC09-011 | PEMR17931 | HF547780 | HF547739 | HF547702 | HF547664 |
| *Meroles* | *ctenodactylus* | AMB4632 | — | HF547782 | — | — | — |
|  |  | JM03609 | — | HF547783 | HF547741 | HF547704 | — |
|  |  | JM03611 | — | — | HF547742 | HF547705 | HF547665 |
|  |  | JM03613 | — | HF547784 | HF547743 | HF547706 | HF547666 |
| *Meroles* | *cuneirostris* | WP921 | — | HF547788 | HF547747 | HF547710 | HF547670 |
|  |  | WP914 | — | HF547787 | HF547746 | HF547709 | HF547669 |
|  |  | MB20484 | MB20484 | HF547785 | HF547744 | — | HF547667 |
|  |  | MCZA38244 | MCZA38244 | HF547786 | HF547745 | HF547708 | HF547668 |

| *Meroles* | *knoxii* | H6179 | H6179 | HF547790 | HF547750 | HF547712 | — |
| --- | --- | --- | --- | --- | --- | --- | --- |
|  |  | AMB5629 | — | DQ871146^$^ | HF547748 | DQ871204^$^ | HF547671 |
|  |  | ATTKMK2 | — | HF547789 | HF547749 | HF547711 | HF547672 |
|  |  | SER017 | — | HF547791 | HF547751 | — | — |
|  |  | SVN084 | PEMR18357 | HF547792 | HF547752 | HF547713 | — |
| *Meroles* | *reticulatus* | WP010 | — | HF547795 | HF547754 | HF547715 | HF547674 |
|  |  | AMB7032 | — | HF547793 | — | — | — |
|  |  | WC09-005 | PEMR17938 | HF547794 | HF547753 | HF547714 | HF547673 |
|  |  | WP011 | — | HF547796 | HF547755 | — | HF547675 |
| *Meroles* | *suborbitalis* | WP967 | — | HF547801 | HF547760 | — | — |
|  |  | AJC638 | — | HF547797 | HF547756 | HF547716 | HF547676 |
|  |  | MB20609 | PEMR16974 | HF547798 | HF547757 | — | — |
|  |  | MB21589 | — | HF547799 | HF547758 | HF547717 | HF547677 |
|  |  | SVN049 | PEMR18376 | HF547800 | HF547759 | HF547718 | HF547678 |
| *Pedioplanis* | *burchelli* | KTH137 | — | DQ871122^$^ | — | DQ871180^$^ | — |
|  |  | MH0334 | — | DQ871120^$^ | HF547761 | DQ871178^$^ | HF547679 |
| *Pedioplanis* | *inornata* | ABE-393-mu | NHMW 35340:9 | DQ871137^$^ | HF547762 | DQ871195^$^ | HF547680 |
|  |  | KTH595 | — | DQ871140^$^ | — | DQ871198^$^ | — |
| *Pedioplanis* | *lineoocellata lineoocellata* | ABA-20-mu | NHMW 35360:1 | DQ871106^$^ | HF547763 | DQ871164^$^ | HF547681 |
| *Pedioplanis* | *lineoocellata pulchella* | MH0336 | — | DQ871107^$^ | HF547764 | DQ871165^$^ | — |
|  |  | SVN189 | — | HF547802 | HF547765 | HF547719 | HF547682 |
| *Pedioplanis* | *namaquensis* | AMB4541 | — | DQ871099^$^ | HF547766 | DQ871157^$^ | HF547684 |
|  |  | AMB4558 | CAS 200033 | DQ871101^$^ | HF547767 | DQ871159^$^ | HF547685 |
| *Tropidosaura* | *gularis* | EL036 | — | HF547803 | — | HF547720 | HF547686 |
|  |  | RSP200 | — | HF547804 | HF547768 | HF547721 | HF547687 |
| *Tropidosaura* | *montana montana* | HB082 | — | HF547805 | HF547769 | HF547722 | HF547688 |
| *Tropidosaura* | *montana rangeri* | MBUR00544 | — | HF547806 | HF547770 | HF547723 | HF547689 |
|  |  | MBUR00552 | — | HF547807 | HF547771 | HF547724 | HF547690 |

* N/A = Individuals were measured alive in the field and released, no voucher specimen deposited in a museum; TM = Ditsong museum; PEM = Port Elizabeth Museum; MCZ = Museum of Comparative Zoology, Harvard University; CAS = Californian Academy of Science; H = Ellerman Collection of Stellenbosch University

$ Makokha JS, et al. (2007) Mol. Phylogenet. Evol. 44 (2), 622-633
