# Supplementary material for: Convergent Evolution Associated with Habitat Decouples Phenotype from Phylogeny in a Clade of Lizards
Source: PLoS One. 2012 Dec 12;7(12):e51636. doi: 10.1371/journal.pone.0051636 (PMC3520956; doi:10.1371/journal.pone.0051636)
Supplement: Table S2 — List of specimens used in morphometric analyses, genus and species names, ID numbers from either the Ditsong museum (TM), Port Elizabeth Museum (PEM), or field trips. (DOCX) [file pone.0051636.s007.docx]

**Table S2: List of specimens used in morphometric analyses, genus and species names, ID numbers from either the Ditsong museum (TM), Port Elizabeth Museum (PEM), or field trips.**

| **Genus** | **Species** | **Number of individuals** | **Museum accession number** | **Field numbers** |
| --- | --- | --- | --- | --- |
| *Australolacerta* | *australis* | 11 | TM56019 | AA02, AA03, AA04, AA06, AA07, AA09, AA10, AA11, AA12, GW08 |
| *Australolacerta* | *rupicola* | 18 | TM53553, TM53554, TM62696, TM62700, TM62701, TM62703, TM62704, TM62705 | FP310, HZ273, HZ278, HZ283, HZ284, T521, T522, T523, T524, T526 |
| *Ichnotropis* | *capensis* | 18 | TM2201, TM2206, TM2209, TM2211, TM2213, TM4749, TM4783, TM4784, TM26871, TM26874, TM26875, TM31076, TM31077, TM31078, TM31304, TM38337, TM62754, TM79735 | None |
| *Ichnotropis* | *squamulosa* | 36 | TM2293, TM4355, TM4552, TM4638, TM4639, TM14524, TM16822, TM16823, TM16824, TM16825, TM21542, TM21543, TM25606, TM30668, TM30823, TM30824, TM30825, TM34405, TM36061, TM61477, TM61478, TM61481, TM61485, TM61486, TM61500, TM61503, TM61516, TM61527, TM61529, TM61548, TM63137, TM80834, TM80835, TM80836, TM80837, TM80841 | None |
| *Meroles* | *anchietae* | 26 | PEMR15913, PEMR15914, PEMR15915, PEMR17286 | WP003, WP004, WP005, WP006, WP323, WP326, WP327, WP913, WP915, WP916, WP917, WP918, WP919, WP925, WP926, WP928, WP929, WP930, WP931, WP933, WP934, WP935 |
| *Meroles* | *ctenodactylus* | 17 | PEMR526, PEMR2155, PEMR2156, PEMR2157, PEMR7403, PEMR11893, PEMR15810, PEMR15813, PEMR15924, PEMR16799, PEMR16800, PEMR17723, TM15772, TM15776, TM15779, TM15780, TM20986 | None |
| *Meroles* | *cuneirostris* | 39 | PEMR2083, PEMR2084, PEMR2085, PEMR2086, PEMR2087, PEMR4848, PEMR6119, PEMR7422, PEMR7425, PEMR7426, PEMR7427, PEMR7460, PEMR7461, PEMR7463, PEMR7500, PEMR7531, PEMR7544, PEMR7557, PEMR7558, PEMR11948, PEMR15921, PEMR15922, PEMR15923, PEMR17288, PEMR17290, PEMR17291 | WP001, WP320, WP322, WP324, WP325, WP912, WP914, WP920, WP921, WP923, WP924, WP936, WP937 |

| *Meroles* | *knoxii* | 80 | PEMR525, PEMR529, PEMR2259, PEMR2260, PEMR2265, PEMR3496, PEMR5784, PEMR6711, PEMR6712, PEMR6713, PEMR6736, PEMR6737, PEMR7001, PEMR7077, PEMR7188, PEMR7545, PEMR7549, PEMR15873, PEMR15916, PEMR15917, PEMR15918, PEMR15919, PEMR15920, PEMR16797, PEMR16798, PEMR17233, PEMR17730, PEMR18351, PEMR18357, PEMR18379, | 2669, FB288, FB366, FB379, FB380, FB440, FB441, FB526, FB619, H3883, H3888, H3889, H6160, H6163, H6177, H6178, H6179, JM03397, JM03398, JM03401, JM03402, JM03403, JM03404, JM03405, SER097, SER098, SER100, SER101, SER105, SER106, SER107, SER108, SER109, SER111, SER112, SER113, SER114, SER115, SER116, SER117, SER119, SER121, SER122, SER123, SER124, SER132, SER135, SER136, SER137, SER149 |
| --- | --- | --- | --- | --- |
| *Meroles* | *reticulatus* | 25 | PEMR2015, PEMR2016, PEMR2017, PEMR2018, PEMR15949, PEMR15954, PEMR15956, PEMR15958, PEMR15959, PEMR15960, PEMR15963, PEMR15964, PEMR15965, PEMR15966, PEMR15967, PEMR17938, TM23959, TM23960, TM23962, TM23963, TM23990, TM23991 | WP010, WP978, WP980 |
| *Meroles* | *suborbitalis* | 61 | PEMR2100, PEMR2101, PEMR2103, PEMR2106, PEMR2107, PEMR2108, PEMR2109, PEMR2110, PEMR2112, PEMR3694, PEMR3696, PEMR4319, PEMR4320, PEMR4344, PEMR4345, PEMR4702, PEMR4734, PEMR4736, PEMR4737, PEMR4738, PEMR4747, PEMR4754, PEMR5065, PEMR6123, PEMR6737, PEMR7483, PEMR7484, PEMR7486, PEMR7488, PEMR11894, PEMR11919, PEMR11944, PEMR11946, PEMR15927, PEMR15928, PEMR15929, PEMR15930, PEMR15931, PEMR15932, PEMR15934, PEMR15938, PEMR15939, PEMR15940, PEMR15941, PEMR15942, PEMR18307, PEMR18376 | WP012, WP013, WP014, WP015, WP016, WP017, WP963, WP966, WP967, WP968, WP969, WP970, WP971, WP976 |
| *Pedioplanis* | *burchelli* | 14 | TM39736, TM39737, TM39739, TM61407, TM61409, TM61410, TM61411, TM80074, TM80083, TM39738, TM39740, TM61408, TM80071, TM80075 | None |
| *Pedioplanis* | *inornata* | 18 | None | WP939, WP940, WP942, WP943, WP944, WP945, WP946, WP947, WP948, WP949, WP950, WP951, WP952, WP953, WP954, WP955, WP956, WP962 |
| *Pedioplanis* | *lineoocellata lineoocellata* | 61 | PEMR2128, PEMR2129, PEMR2138, PEMR2139, PEMR4415, PEMR4416, PEMR4742, PEMR4846, PEMR4847, PEMR10504, PEMR10506, PEMR10507, PEMR10508, PEMR10509, PEMR10511, PEMR10513, PEMR10514, PEMR10515, PEMR10516, PEMR10517, PEMR10527, PEMR10528, PEMR10529, PEMR10530, PEMR10531, PEMR10532, PEMR10533, PEMR10534, PEMR10632, PEMR10669, PEMR10670, PEMR10673, PEMR10674, PEMR10675, PEMR16865, PEMR16868, PEMR16869, PEMR18236, PEMR18252, PEMR18265, PEMR18286, PEMR18287, PEMR18288, PEMR18289, PEMR18297, PEMR18298, PEMR18304, TM4347, TM4348, TM4349, TM4350, TM4351 | None |
| *Pedioplanis* | *lineoocellata pulchella* | 30 | PEMR4415, PEMR4416, PEMR6651, PEMR6652, PEMR6660, PEMR6662, PEMR6663, PEMR6664, PEMR6668, PEMR6669, PEMR6757, PEMR7063, PEMR7097, PEMR7104, PEMR8562, PEMR11220, PEMR11228, PEMR11258, PEMR11259, PEMR17231, PEMR17238, PEMR17265, PEMR17555, PEMR17856, PEMR17857, PEMR17880, PEMR17882, PEMR18230, PEMR18232, PEMR18369 | None |
| *Pedioplanis* | *namaquensis* | 18 | TM14496, TM14497, TM25701, TM25717, TM26986, TM26987, TM27013, TM37682, TM37764, TM38942, TM49193, TM53589, TM54300, TM54317, TM54636, TM56418, TM63154, TM71483 | None |
| *Tropidosaura* | *gularis* | 10 | TM19959, TM20174, TM20176, TM20215, TM20293, TM20294, TM20295, TM39734, TM39735, TM52522 | None |
| *Tropidosaura* | *montana montana* | 5 | TM55618, TM55619, TM55620, TM56034, TM56035 | None |
